# Expression and RNA Interference of Salivary Polygalacturonase Genes in the Tarnished Plant Bug, *Lygus lineolaris*

**DOI:** 10.1673/031.010.14133

**Published:** 2010-10-06

**Authors:** William B. Walker, Margaret L. Allen

**Affiliations:** ^1^ USDA-ARS, Biological Control of Pests Research Unit, 59 Lee Road, NBCL, Building 8, Stoneville, Mississippi; ^2^ Current address: The Swedish University of Agricultural Sciences, Department of Plant Protection Biology, Division of Chemical Ecology. Alnarp, Sweden

**Keywords:** extraoral digestion, gene expression, gene knockdown, RNAi, salivary enzyme

## Abstract

Three genes encoding polygalacturonase (PG) have been identified in *Lygus lineolaris* (Palisot de Beauvois) (Miridae: Hemiptera). Earlier studies showed that the three *PG* gene transcripts are exclusively expressed in the feeding stages of *L. lineolaris.* In this report, it is shown that all three transcripts are specifically expressed in salivary glands indicating that PGs are salivary enzymes. Transcriptional profiles of the three *PGs* were evaluated with respect to diet, comparing live cotton plant material to artificial diet. *PG2* transcript levels were consistently lower in cotton-fed insects than those reared on artificial diet. RNA interference was used to knock down expression of *PG1* mRNA in adult salivary glands providing the first demonstration of the use of this method in the non-model insect, *L. lineolaris.*

## Introduction

The tarnished plant bug, *Lygus lineolaris* (Palisot de Beauvois) (Heteroptera: Miridae) is a pest that has a broad host range, including several major crops such as cotton and corn, as well as many native plants (Esquivel and Mowery 2007). Nymphs and adults feed on the flowers and fruits of many plants causing abscission and deformation of both (Strong 1970). The insects feed by inserting haustellate mouthparts into plant tissue, injecting salivary enzymes, and then ingesting the liquefied plant material. This is referred to as extraoral digestion, piercing-sucking, and/or “lacerate and flush feeding” (Wheeler 2001). With regard to current technology, this mechanism of feeding makes the pest difficult to control; transgenic crops incorporating crystal toxins from *Bacillus thuringiensis* (Bt) do not affect *L. lineolaris*, and resistance to chemical pesticides is reported in pest populations (Snodgrass 1996). Thus, *L. lineolaris* has emerged recently as an economically relevant cotton pest.

Multiple forms of polygalacturonases (PG), enzymes which catalyze hydrolysis of α-1,4-glycosidic linkages in polygalacturonic (pectic) acid, have been detected in Lygus plant bug saliva biochemically (Strong and Kruitwagen 1968; Laurema et al. 1985; Agblor et al. 1994; Frati et al. 2006; Celorio-Mancera et al. 2009) and DNA encoding of three unique PGs have been cloned (Allen and Mertens 2008). Recently, it has been confirmed that PG enzymatic activity is responsible for plant damage caused by the Lygus plant bug; active PG enzymes, when injected into plant tissue (Shackel et al. 2005; Celorio-Mancera et al. 2008), induce plant damage previously described and prescribed to salivary gland enzymatic activity (Strong 1970). PG enzymes are common in many species of fungi in multiple forms (Niture 2008) and are associated with fungal pathogenicity. PG proteins degrade the pectin substrate with different enzymatic activities, and thus multiple polymorphic enzymes serve a logical use to organisms that must degrade pectin, which is a highly polymorphic complex carbohydrate, as part of an insect feeding strategy. Polygalacturonase-inhibiting proteins (PGIPs) are present in plants, and serve as defense against pathogenic fungi and insects (D'Ovidio et al. 2004; Frati et al. 2006). These PGIPs are also numerous and vary in activity. It follows that a better understanding of the PGs produced by Lygus plant bug pests, and the PG/PGIP interactions during the insect (or fungus) and plant interaction should lead to identification of methods for mitigation of plant damage through *PGIP* gene manipulation or selection.

**Figure 1.**
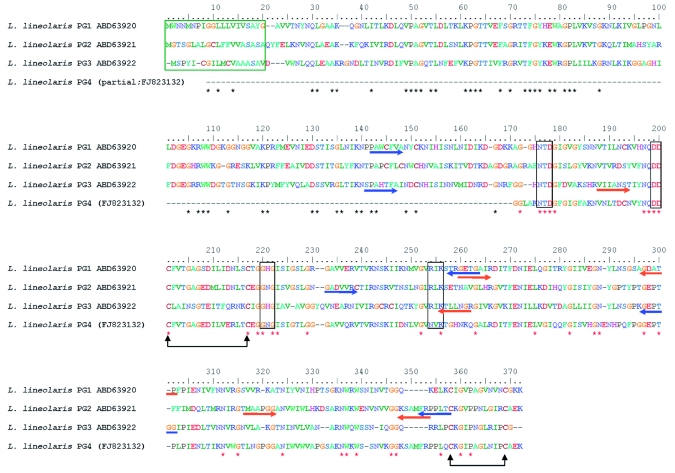
Multiple Sequence Alignment and primer locations of Lygus *lineolaris* deduced PG amino acid sequences. * indicates conserved residues in all sequences. Predicted N terminal signal peptide and enzymatically critical amino acid motifs are boxed. Nucleotide sequences used as primers are indicated by arrows pointing the direction of amplification. Primer pairs used for generation of the dsRNA construct are blue. Primer pairs used for qRT-PCR amplification are orange. Linked arrows pointing upward indicate putative cysteine disulfide bridges. All sequences except for *L. lineolaris* PG4 are derived from full length cDNA information. Sequence alignment was generated with the ClustalW2 program (Larkin et al. 2007). High quality figures are available online.

While PG enzymes have been isolated from Lygus plant bug salivary glands, and multiple forms were shown to be present and active (Celorio-Mancera et al. 2008; CelorioMancera et al. 2009), it has never been conclusively shown that the three *PG* genes cloned and identified from *L. lineolaris* are of salivary gland origin and whether PGs are transcribed in other digestive tissues. The current study clearly shows that all three of the previously identified *L. lineolaris PG* genes are expressed in salivary glands primarily, if not exclusively. Additionally, this study shows that gene expression is transcriptionally regulated in the insect based on diet for one of the known PGs and the three genes vary in their susceptibility to RNA interference (RNAi) gene knockdown. A fourth *L. lineolaris PG* gene (Accession number FJ823132, Figure 1.) has been identified, but not yet cloned in its entirety and was not analyzed in this report. These studies further illustrate the complexity of this important gene family, and highlight the difficulty this type of polyphagous insect poses to crop protection scientists and farmers alike.

## Methods and Materials

### Insect Handling and Dissections

Insects used for all studies were laboratory reared at 60% RH, 16:8 L:D, with 23.5° C daytime temperature and 17° C night temperature (Allen 2007). For the food source experiments, insects were provided with fresh cotton sprigs (*Gossypium hirsutum*) or artificial diet food packets (Cohen 2000) as fifth instar nymphs, and were allowed to feed for 4–5 days. After this period, the insects had matured to the adult stage and were collected as adults. Insects were removed from the plants or artificial diet and held starved for one hour prior to dissection to promote a more consistent state of salivary activity.

For tissue RNA extractions, adult insects were dissected in phosphate-buffered saline by cutting off the terminal abdominal segment, then pulling the head and prothorax away from the remaining segments. In this manner, salivary glands and the alimentary system were removed from the insect and then separated. The legs were removed from the remaining body. Thus, the salivary glands, guts, body carcass, and legs were separately placed in collection tubes for RNA extractions destined for tissue-specific reverse transcription PCR (RT-PCR)/(cDNA synthesis); the heads were discarded. Care was taken to verify that no gut was included with salivary gland tissue, and vice versa, and that neither gut nor salivary gland remained in the body cavity. Processing the legs as tissue sample was an extra measure of caution to rule out contamination of the body cavity extractions with gut or salivary gland tissue. For RNAi knockdown and host plant experiments, head/pronotum portions, and some gut portions were collected together with the salivary glands. Twelve or more insects were pooled for tissue samples and used in tissue-specific RT-PCR and food source experiments; for RNAi knockdown experiments five insects were pooled per sample.

### RNA Extraction and cDNA Synthesis

Total RNA was extracted from live insects and freshly dissected insect tissue using USB (Cleveland, OH) PrepEase™ kits following manufacturer instructions. Under this protocol, removal of contaminant genomic DNA is performed with on-column DNase digestion. Yield and purity estimates were measured with a NanoDrop™ spectrophotometer (www.nanodrop.com). After total RNA extraction each sample was diluted with deionized water to 200 ng/µl, then 1.5 µg of tRNA was used as the source for first strand cDNA synthesis using materials from the Cells-to-cDNA II kit (Ambion, www.ambion.com): Oligo dT(18) primers (6.67 µM, final concentration), MMLV reverse transcriptase (100 U/µl), and an RNase inhibitor (20 U/µl). Enzymatic incubation was carried out at 42 °C for 60 min. For all samples NoRT controls were generated in which no reverse transcriptase was added to the reaction mixtures.

### RNA Interference

For injection of dsRNA, 4th–5th instar nymphs were isolated from the laboratory colonies and kept in 100 × 15mm Petri dishes (Fisher Scientific, www.fishersci.com) with free access to clover leaves. Adults were isolated as they eclosed and allowed free access to alfalfa leaves. In all cases plant materials originated from plants grown in an in-house laboratory greenhouse and were cultivated pesticide-free. For nymphs and adults plant materials were changed every 24 h. Prior to injections adults were chilled for 5 min at 4° C, then anesthetized with carbon dioxide gas and placed in between a parafilm sandwich, which consists of two square sheets of parafilm, in which insects are aligned for injection on top of the fully taught, unstretched piece, and then covered and immobilized with the other piece which is fully stretched and pressed on top of the insects.

Male and female tarnished plant bug adults were isolated and injected within 48 hours of eclosion. Control injection groups were injected with 1X Phosphate Buffered Saline (PBS), pH 7.4, 1% blue food coloring, or eGFP dsRNA. Experimental injection groups were injected with PG1, PG2, or PG3 dsRNA. All insects were injected with Femto-Tip injection needles (Eppendorf, www.eppendorf.com; 0.5 µm i.d., 0.7 µm o.d.) in the abdomen using a Femto-Jet® (Eppendorf) microinjector with an average volume of 1 µl of injection fluid. For the dsRNA treatment 300 ng to 400 ng of dsRNA reconstituted in 1X PBS was injected. Doublestranded RNA was prepared using ABI (Ambion) MEGAscript® transcription kit following manufacturer instructions. dsRNA template sequence information is shown in Figure 1.

After injections insects were released from the parafilm and placed in clean Petri dishes with free access to fresh alfalfa leaves, and kept at standard rearing conditions. For all injected insects, the survival rate was 57%–77% at 72 h post-injection, at which time insects were dissected over a frozen ice block for the total RNA extraction procedure.

### Semi-Quantitative PCR

PCR reactions were performed in an MJ Research (www.mjr.com) thermal cycler, using program settings appropriate for proper primer annealing and expected amplicon extension, over a period of 30 cycles, and following recommendations from Clontech (www.clontech.com) provided with the Advantage® 2 polymerase mix. See Table 1 for specific parameters. All primers were designed with the use of MFold (Zuker 2003), Primer 3 (Rozen and Skaletsky 2000), and IDT OligoAnalyzer (Integrated DNA Technologies, www.idtdna.com) web-based software. PCR primers are listed in Table 1.

### Quantitative Real Time PCR

Quantitative Real Time PCR (qRT-PCR) experiments were carried out with an MJ Mini Opticon thermocycler and MJ Opticon Monitor software (BioRad, www.biorad.com). For all reactions, the following contents were added: 1 µl of cDNA sample, 12.5 µl of enzymatic mix, iTaq SYBR Green supermix with ROX (BioRad, Hercules, California), 6.5 µl water, and 5 µl of gene specific primers (100 nM final concentration of each primer) for a final reaction volume of 25 µl. For the food source related PG expression analyses, the data were analyzed using the Pfaffl equation (Pfaffl 2001). For the RNAi knockdown experiments, the data were analyzed with geNORM software (Vandesompele et al, 2002) utilizing C_t_ values and amplification efficiencies as a basis for Delta-Delta-Ct comparison. Final relative expression levels across experimental and control groups were determined using geNORM generated normalization factors, which were derived from the expression analysis of five control genes and applied to Delta-Ct values. For all reactions, the PCR amplification protocol was as follows:

**Table 1.**
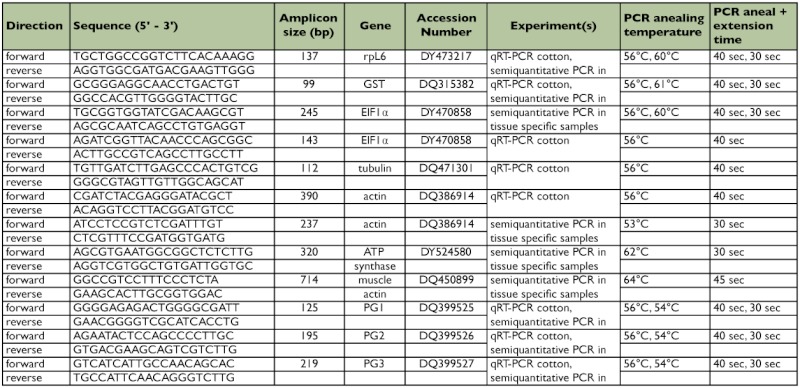
Primers used to amplify *Lygus lineolaris* cDNA

**Table 2.**
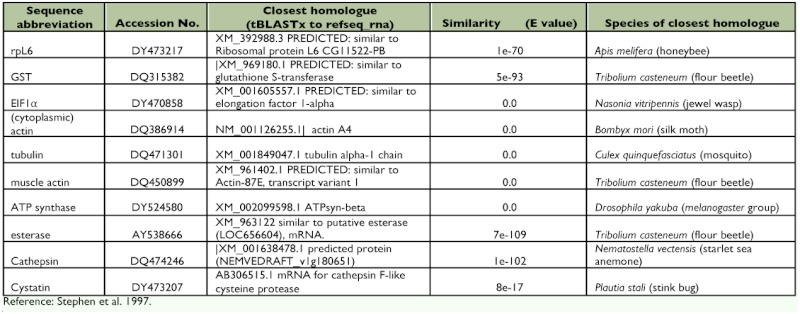
*Lygus lineolaris* sequences chosen as control genes for expression studies.

Initiation Phase - 95° C for 3 min; Amplification Phase - 95° C for 10 s, 56° C for 40s; Repeat amplification phase 39 times; Melting Curve Phase - 40° C to 95° C gradient, with analysis every 1.0° C. For all samples melting curves were analyzed to verify the nature/quality of amplification products. Primers are listed in Table 1, amplification regions are indicated in Figure 1, and descriptions of the control sequences are included in Table 2.

## Results

**Figure 2.**
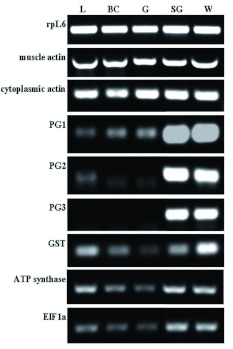
*Lygus lineolaris* PG genes are predominantly expressed in salivary gland tissue. Spatial expression patterns of PG genes in adult *L. lineolaris*. Semi-quantitative analysis of PG1, PG2, and PG3 expression in leg (L), body cavity (BC), gut (G), salivary gland (SG), and whole insect (W) samples. Expression of several control genes (rpL6, muscle actin, cytoplasmic actin, GST, ATP synthase, ElF1a) in this context provides a basis for sample tissue integrity, thus giving validity to PG expression profiles. High quality figures are available online.

To verify that the cloned cDNA sequences generated for PG1, PG2, and PG3 (Allen and Mertens 2008) were responsible for encoding enzymes in the salivary glands, expression profiles of these genes were examined. Total cDNA, prepared from pooled samples including parts from at least twelve insects, were analyzed with semi-quantitative PCR. Because each tissue sample was pooled from several insects, and equivalent starting amounts of total RNA were used for cDNA synthesis, each amplification reaction represented an equal quantity of a given transcript proportionate to the total RNA sample. Multiple control genes were chosen in anticipation that several of them would be expressed constitutively throughout all life stages and all tissue samples of the insect. These controls were amplified alongside the *PG* genes and the varying expression levels of the different control genes were interpreted as a good indication that our results were consistent with actual expression levels in the organism. Consistently strong expression of the control genes *rpL6, muscle actin,* and *cytoplasmic actin,* and moderate expression in all samples of the control genes *GST, ATP synthase,* and *ElF 1a* in all tissues were clearly differentiated from the strong amplification of all the three *PG* genes only in salivary gland samples and whole insect samples (Figure 2).

Previous research indicated a large amount of individual variability in *PG* gene transcription (Allen and Mertens 2008). Having verified that all three *PGs* were transcribed primarily in the salivary gland tissues, transcriptional variation between insects feeding on artificial diet or cotton plants was examined. The experiment was performed three times and qRT-PCR data were analyzed with the Pfaffl equation (Pfaffl 2001), which quantifies relative expression levels of the target gene across samples normalized by control gene expression data. Several control genes were chosen for singular normalizations, and for each target gene relative expression ratios were averaged incorporating data for all control gene analyses. Results are shown in Table 3.While the third experiment indicated upregulation of *PG1* and little change from control in *PG3,* overall results indicate downregulation *of PG* genes when feeding on cotton compared to the laboratory diet developed as an ideal food source (Cohen 2000). Specifically, *PG2* expression was downregulated in *L. lineolaris* that had fed on cotton in all three experimental replicates. All control genes used for comparison of the *PG* expression levels are included in Table 3; some of these genes could have been regulated in response to diet, however, the results do not clearly indicate this. A description of the sequences used as controls is included in Table 2.

**Figure 3.**
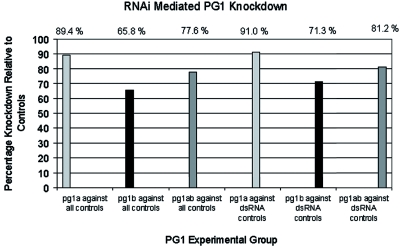
RNAi mediated knockdown of PG1. Percentage knockdown of PG1 mRNA in PG1 injected groups relative to various control injection groups. In all cases, PG1 expression is normalized to 5 control gene expression levels (described in methods and results sections). Experimental groups are compared individually (solid bars) and combined (dashed bars) to the control groups. “All controls” include those injected with eGFP dsRNA as well as injection buffer constituents alone, where as “dsRNA controls” consist of this solely injected with eGFP dsRNA.Ratio of PG expression in treated (cotton-fed) vs. control (artificial diet) adult *Lygus lineolaris.* High quality figures are available online.

In order to glean functional roles of the *PG* genes in feeding and digestive processes in *L. lineolaris,* the RNA interference technique (Fire et al. 1998) was utilized to knock down *PG1, PG2,* and *PGS* transcript levels. dsRNA templates were amplified with the intent of incorporating at least one enzymatically active core amino acid sequence. The actual positions of the PG open reading frames amplified to produce the dsRNA templates are shown in Figure 1. qRT-PCR assays were performed to assess *PG* expression levels in experimental groups relative to controls. For these experiments, samples from PG1, PG2, and PG3 dsRNA injection groups were independently compared against samples from five injection control groups, which included two injection buffer control groups and three dsRNA control groups, all derived from insects injected with eGFP dsRNA. For all samples *PG* expression levels were normalized collectively against the expression levels of five control genes with the geNORM software (Vandesompele et al. 2002). Figure 3 illustrates RNAi induced knockdown of *PG1* expression levels in *L. lineolaris* bugs that were injected with PG1 dsRNA. An average of 77.6% knockdown of PG1 expression levels in PG1 dsRNA derived sample groups was observed compared to all control groups, as well as a 81.2% knockdown as compared to the eGFP dsRNA injected control groups. Knockdown of *PG2* and *PG3* was not observed (data not shown).

**Table 3.**
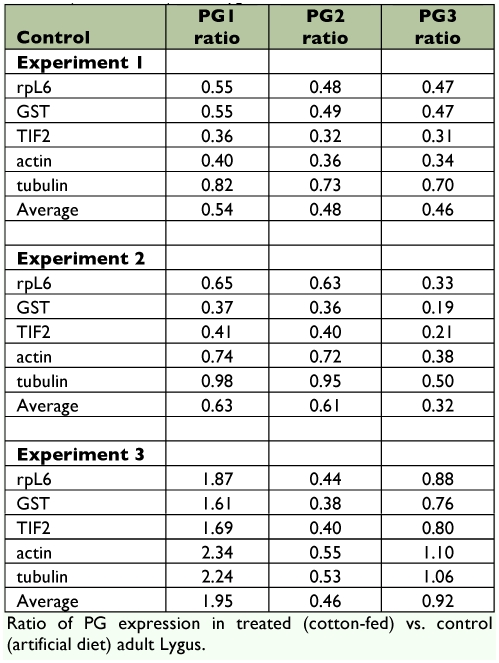
Ratio of PG expression in treated (cotton-fed) vs. control (artificial diet) adult *Lygus lineolaris.*

## Discussion

Biochemical activities of Lygus plant bug saliva have been reported recently. Salivary isolates taken directly from insects (Celorio-Mancera et al. 2008) exhibit pectin-degrading activity, and plant damage essentially identical to feeding damage was produced by mechanical microinjection of Lygus plant bug saliva (Shackel et al. 2005). However, PG activity was also detected in guts of Lygus plant bugs (Agusti and Cohen 2000), and the tissue in which the three cloned *L. lineolaris PG* genes were transcribed was not conclusively demonstrated previously.

When multiple genes encoding polygalacturonases were found among *L. lineolaris* ESTs (Allen 2007), their possible redundancy immediately raised questions about regulation and specificity. When *L. lineolaris* fed on different host plants were some *PG* genes downregulated and others upregulated? Substantial differences in protein profiles were reported from collections of *L. hesperus* saliva when exposed to different host substrates (Habibi et al. 2001). If one of the *PG* genes were to be incapacitated by RNA interference or inhibition would other forms serve as alternative digestive mechanisms, making detection of loss of function difficult or impossible? Alternatively, is each PG unique and necessary for the overall digestive process? We hypothesized the former, which was supported when insects injected with PG1 dsRNA displayed no obvious phenotype. There was no apparent decrease in longevity of insects injected with double-stranded PG1 (results not shown) compared to controls. RNAi has been suggested as a plant-incorporated pesticide strategy (Baum et al. 2007; Mao et al. 2007), but clearly *PG1* alone is not a candidate gene. A combinatorial approach, using RNAi to target multiple *PG* genes may yield greater insights towards the specific roles of each factor if such a distinction beyond redundancy exists. Furthermore, precise discernment is contingent upon a more comprehensive understanding of the digestive enzyme components in *L. lineolaris* saliva. The recent identification of a fourth *L. lineolaris PG* gene suggests this knowledge is far from complete.

The RNA interference technique has been widely employed within the field of insect molecular biology (Price and Gatehouse 2008). RNAi has been reported as a viable mechanism of molecular genetic analysis in several insect orders including other Hemipterans (Araujo et al. 2006; Ghanim et al. 2007; Hrycaj et al. 2008). This article represents the first report on RNAi mediated knockdown in the agricultural pest, *L. lineolaris.* Further work, however is necessary to determine if practical RNAi based approaches will be feasible in mitigating economic damage caused by *L. lineolaris* bugs.

Preliminary experiments (not shown) using multiple food sources indicated dramatic but inconsistent variation in *PG* expression by individual insects consistent with the results reported in previous research (Allen and Mertens 2008). A simplified experimental plan was carried out, comparing one economically relevant food source, cotton, with standard experimental rearing diet (Cohen 2000). Cotton produces compounds known to be toxic to insects and that deter insect feeding (Bottger et al. 1964), including PGIPs (Shi et al. 2009), and yet *L. lineolaris* readily feeds on cotton. Therefore, upregulation of salivary PG transcripts was anticipated. The artificial diet contains a mixture of plant and meat derived components, of which the plant materials (including toasted wheat germ, lima bean meal, and soy flour) are mixed and autoclaved. It is suggested (Cohen 2000) that artificial diet is composed to facilitate extraoral digestion which would be mediated by secretion of salivary gland enzymes, including the PGs. Surprisingly, two out of the three experiments demonstrated downregulation of all three *PG* forms, and only the third experiment displayed upregulated *PG1.* The overall results of these experiments only clearly identified *PG2* as consistently affected by feeding on cotton as a host, and the transcription was down-regulated. Speculatively, the plant cell wall pectin components of cultivated cotton may be relatively easy to digest for *L. lineolaris.* This could partially explain the pest relationship of *L. lineolaris* to cotton.

Expression of functional *L. lineolaris* PGs in heterologous systems has been unsuccessful thus far, so it has been impossible to identify specific PG activities for the various enzyme forms. Experimental evidence has indicated both endo- and exo- polygalacturonase activities in *L. hesperus* saliva (Celorio-Mancera et al. 2009), and the same are certainly expected in *L. lineolaris.* When a more complete set of PGs and other salivary enzymes are isolated from both species of Lygus plant bugs we hope to use this information to identify traits and genes useful for crop defense against Lygus plant bug damage.

## Abbreviations

PG: polygalacturonase;
EST: expressed sequence tag;
PGIP: polygalacturonase inhibiting protein;
RNA: ribonucleic acid;
RNAi, RNA: interference;
cDNA: complementary 2-deoxyribonucleic acid;
PCR: polymerase chain reaction;
RT-PCR: Reverse Transcription PCR;
qRT-PCR: quantitative Real Time PCR
